# Single Dose of Levofloxacin versus Three Dosages for Prophylaxis in Prostate Biopsy

**DOI:** 10.1155/2014/875670

**Published:** 2014-09-03

**Authors:** Edgar Linden-Castro, Marcela Pelayo-Nieto, Alejandro Alias-Melgar, Fernando Carreño-de la Rosa

**Affiliations:** Urology Department, Centro Medico Nacional “20 de Noviembre” México, Félix Cuevas 540, Del Valle, Benito Juárez, 03100 Ciudad de México, DF, Mexico

## Abstract

Transrectal ultrasound-guided core prostate biopsy is a key event in the diagnosis of prostate cancer, transient side events such as local pain, haematuria, haematospermia, dysuria, and rectal bleeding are reported in a large number of patients. Antimicrobial agents lower the incidence of postbiopsy infectious complications. The timing and duration of the regimen and the route of administration remain controversial. We developed a standard prophylactic regimen, in which safety and efficiency were maximized, while costs and variability were minimized. Accordingly we prospectively evaluated 425 consecutive patients, who underwent outpatient transrectal ultrasound-guided prostate biopsy after a single dose versus three doses of levofloxacin.

## 1. Introduction

Prostate biopsy guided by transrectal ultrasound is a key element in the diagnosis of prostate cancer, but its implementation lacks in terms of safety and complications [1]. The presence of adverse events, such as local pain, hematuria, hematospermia, dysuria rectal bleeding, prostatitis, epididymitis, orchitis, and sepsis, is reported in many patients; 20–50% have bacteriuria, 3–10% had fever with lower urinary tract symptoms, and the incidence of sepsis in patients undergoing this procedure is 0.5–5% [2–6]. Antimicrobial agents reduce postinfectious biopsy complications [7, 8]. Despite performing more than one million prostate biopsies in the United States and Europe each year and although clinical guidelines exist, there is no consensus on the regime of antimicrobial prophylaxis in clinical practice. Some studies recommend the use of enemas before biopsy, but other groups question their benefit [9].

Antimicrobial prophylaxis is universally accepted and multiple points of view regarding the time of drug administration, duration, and medication delivery exist [10]. Most studies use schemes ranging from prophylaxis use previous hours, to the use of antimicrobials 1–3 days before the procedure [11]. In the present study we evaluate and compare the efficacy of single dose of 500 mg of levofloxacin orally administered at the day of the procedure versus three doses of the same drug for prophylaxis in patients undergoing transrectal prostate biopsy guided by ultrasound.

## 2. Material and Methods

We evaluated 615 patients with standard indication for prostate biopsy guided by transrectal ultrasound (elevation of prostate specific antigen (PSA), abnormal digital rectal examination). All those patients with hypersensitivity to the drug, indwelling catheter, lower urinary symptoms (dysuria, suprapubic pain, urgency, and urgency), and history of febrile urinary tract infection a month before the procedure, as well as those with a history of acute retention urine and hematuria, were excluded; a total of 425 patients were eligible; patients were randomly divided into two groups using GraphPad Prism 6.0: Group A was composed of 205 patients who were administered a single dose of levofloxacin (500 mg) orally 60–120 minutes before the procedure, because the peak levofloxacin levels within the prostate were reached within one hour after oral administration of this dose and Group B consisted of 220 patients who were administered levofloxacin (500 mg) every 24 hours for two days before and on the day of the procedure. We evaluated the status of these variables: diabetes mellitus, body mass index (calculated as weight in kilograms divided by height in meters squared kg/m^2^) divided into normal weight 18.5–24.9 kg/m^2^; overweight 25.0–29.9 kg/m^2^; 20–34.9 kg/m^2^ obesity grade I; obesity grade II > 35 kg/m^2^ (adapted WHO-2004) [12], and prostate volume. The primary end point is the efficacy of three doses versus single dose, taking into consideration ours variables. All patients underwent bowel preparation with polyethylene glycol orally administered prior to transrectal biopsy day. The biopsy was performed using the equipment Aloka Prosound ultrasound *α*6, with intracavitary transducer 5–10 MHz with needle biopsy BARD 18 Ga-20 cm, obtaining samples of 22 mm. We applied simpre lidocaine in the periprostatic plexus for local anesthesia using a EchoTip Skinny needle chiba Tip 22 Ga/20 inch; 12 cylinders were obtained; 100% of patients underwent urinalysis after procedure but only the patients with a febrile episode underwent a urine culture. Febrile episode of urinary tract was defined as fever ≥ 38.0 degrees Celsius, accompanied by at least one symptom of urinary tract (urgency, frequency, dysuria, and pain suprapubic). Patients with this condition were hospitalized and paraclinical evaluation was complemented with general, urine, and blood culture studies. The aforementioned variables were correlated using Fisher's exact test and Student's *t*-test; statistical analysis was performed using IBM SPSS statistics 19.

## 3. Result

The 425 patients had a prostate volume of 71.14 cc, an average age of 66.86 years (±8.11), and PSA mean of 22.13 ng/mL; they were stratified by BMI, presenting normal weight 19.04%, overweight 54.76%, obesity grade I 20.23%, and obesity grade II 5.95%; in conclusion of this, 80.92% of our patients have some degree of overweight or obesity and 36.90% have diabetes mellitus; then they were randomly divided in two groups. Group A comprised 205 patients that received a single dose of levofloxacin 500 mg orally, with an average age of 66.22 years (±7.95) and PSA mean of 23.07 ng/mL; 43.9% have DM, 4.3% presented febrile urinary tract infection with a positive culture for* E. coli* and* Klebsiella pneumoniae* and two patients had sepsis (0.97%), with average prostate volume of 65.80 cc, and 90.17% in this group had overweight. Group B comprised 220 patients that received three doses of 500 mg levofloxacin orally, with an average age of 67.45 years (±8.31) and PSA mean of 21.26 ng/mL; 31.8% of patients have DM and 4.45% presented febrile urinary tract infection, in the same manner as in Group A; the body predominant in urine culture was* E. coli*; no patients in this group presented sepsis; mean prostate volume was 75.61 cc, of this group of patients; 72.74% had been overweight; in both groups the antibiotic was changed according to the antibiogram. All patients are monitored at 3 weeks of the procedure, and those who had febrile infection and sepsis received two more consultations at 4 and 6 weeks. Both groups are similar and we did not find significant difference in complications (*P* = 0.66) (Table 1); performing analysis considering DM, we did not identify difference in both groups regarding the risk of complications, and also when patients were analyzed with regard to body mass index and risk of complications, in both groups no difference was found (Table 2).

## 4. Discussion

Millions of men undergo prostate biopsies worldwide each year as part of the diagnostic method of prostate cancer detection [13]. Recent retrospective studies in Europe and USA have reported an apparent increase in the incidence of infectious complications after prostate biopsy [14].

Prostate biopsy and prostate specific antigen has low specificity in cancer screening with a detection rate of 22.8–42% in the initial prostate biopsy. A diagnostic test with less than 50% positive detection rate should be as safe as possible [13, 14]. Despite some international recommendations for antibiotic prophylaxis in BTRP, there is no agreement in most centers regarding the use of certain drugs and prophylaxis [15].

Infectious complications occur from 1 to 6% of patients who undergo a prostate biopsy; this includes fever, urinary tract infection, acute prostatitis, and orchiepididymitis [14]. One of the most serious complications associated with BTRP is sepsis, which may jeopardize the patient's life. Fortunately it occurs only in 0.5–1% of patients who undergo BTRP. The frequency of infection varies among studies; most centers report a hospitalization rate of 0–6.3%. Of 72,500 biopsies performed in the UK hospitals 2.15–3.6% were readmitted [15]. The Global Prevalence Study of Infections in Urology shows an incidence of 3.5% and 3.1% in febrile UTI hospitalization after the BTRP [16]. Meanwhile Park et al. reported a low frequency of infectious complications of 0.3% with rectal preparation and 6% without (*P* < 0.001) [15]. A Cochrane review concluded that the use of antibiotics and enema reduces the risk of bacteremia (RR 0.25; 95% CI, 0.08 to 0.75) compared with antibiotic alone, although there was no difference with fever and infection [15].

Different studies have shown the benefit of the use of fluoroquinolone prophylaxis to reduce infectious complications in BTRP [5]. Some studies, showed significant decrease of infectious complications in the use of fluoroquinolones compared with placebo (8% versus 25%) [15]. The concentrations of levofloxacin in prostate fragments obtained by transurethral resection and determined that concentrations were adequate for an effective treatment for the most common pathogens, demonstrating the excellent bioavailability of the drug [16]. They also determined that the peak concentration of levofloxacin was achieved within the first hour after the intravenous administration of a dose of 500 mg orally. Likewise, high plasma concentrations are reached within 30 to 60 minutes after oral administration. Levofloxacin has a half-life of 6 to 8 hours, allowing diary dosage. In our study to assess the increased risk of infection with a dose of levofloxacin versus three doses, there was no significant difference (*P* = 0.66).

In our country the resistance rates tend to be increasing for pathogens like* E. coli*. There has been an increase in resistance to the first-line antimicrobials traditionally used. In the United States there is evidence that the resistance to trimethoprim-sulfamethoxazole is relatively low (17%) and the* E. coli* resistance to fluoroquinolones is only 2.5%, justifying its indication only in complicated urinary tract infection or when the antibiogram shows an advantage. Levofloxacin resistance in our country in the outpatient presents great variability (7.6–29.7%), showing higher resistance rates in hospitalized patients and patients in intensive care units (40–66%).

In 2002, Griffith et al. presented a study with 400 patients which identify DM and steroids use as risk factors for infectious complications in BTRP; in our study there was no significant difference in the administration of one dose versus three doses for patients with DM or in obese patients and because of this we do not consider that diabetic patients should receive a more prolonged prophylaxis.

An important point for future evaluation is the presence of levofloxacin resistance of fecal* E. coli*. One study showed that the presence of fecal* E. coli* strains resistant to levofloxacin represents an important development of infectious complications in BTRP risk [13]. To date there are few randomized studies showing the use of result of rectal swab culture for prophylactic antibiotics use in BTRP [16–18].

## 5. Conclusions

Due to its efficiency and simplicity, a single dose of 500 mg levofloxacin represents excellent choice for prophylaxis in patients undergoing transrectal prostate biopsy. Single dose facilitates patient compliance to prophylactic management, and this dosage can decrease the cost of antimicrobial therapy, with a safety profile similar to three doses effectively.

## Figures and Tables

**Table 1:** Patient characteristics and clinical results.

	1 dose (A)	3 doses (B)	*P* = 0.66
*N*	205	220	
Age	66.22 (±7.95)	67.45 (±8.31)	*P* = 0.26
PSA mean	23.07 ng/mL	21.26 ng/mL	*P* = 0.56
DM	43.90%	31.80%	*P* = 0.57
FUTI	4.30%	4.45%	
Sepsis	0.97%	0	
Volume	65.80 cc	75.61 cc	*P* = 0.24

FUTI: febrile urinary tract infection.

**Table 2:** Details of two patients with sepsis after BTRP.

Patients	Age (y)	Type of infection	Blood culture	Risk factor for the event
1	62	GUI + sepsis	*E. coli *	Advancer CVD and secondary neurologic disorder
2	71	GUI + sepsis	*E. coli *	History of prostatitis, LUTS

GUI: genitourinary infection; LUTS: lower urinary tract symptoms; CVD: cardiovascular disease; BTRP: transrectal prostate biopsy.

## Abbreviations

PSA: Prostate specific antigen
BTRP: Transrectal prostate biopsy
DM: Diabetes mellitus
FUTI: Febrile urinary tract infection.
